# Rapid Recycling of Ca^2+^ between IP_3_-Sensitive Stores and Lysosomes

**DOI:** 10.1371/journal.pone.0111275

**Published:** 2014-10-22

**Authors:** Cristina I. López Sanjurjo, Stephen C. Tovey, Colin W. Taylor

**Affiliations:** Department of Pharmacology, University of Cambridge, Cambridge, United Kingdom; SUNY College of Nanoscale Science and Engineering, United States of America

## Abstract

Inositol 1,4,5-trisphosphate (IP_3_) evokes release of Ca^2+^ from the endoplasmic reticulum (ER), but the resulting Ca^2+^ signals are shaped by interactions with additional intracellular organelles. Bafilomycin A_1_, which prevents lysosomal Ca^2+^ uptake by inhibiting H^+^ pumping into lysosomes, increased the amplitude of the initial Ca^2+^ signals evoked by carbachol in human embryonic kidney (HEK) cells. Carbachol alone and carbachol in combination with parathyroid hormone (PTH) evoke Ca^2+^ release from distinct IP_3_-sensitive Ca^2+^ stores in HEK cells stably expressing human type 1 PTH receptors. Bafilomycin A_1_ similarly exaggerated the Ca^2+^ signals evoked by carbachol or carbachol with PTH, indicating that Ca^2+^ released from distinct IP_3_-sensitive Ca^2+^ stores is sequestered by lysosomes. The Ca^2+^ signals resulting from store-operated Ca^2+^ entry, whether evoked by thapsigargin or carbachol, were unaffected by bafilomycin A_1_. Using Gd^3+^ (1 mM) to inhibit both Ca^2+^ entry and Ca^2+^ extrusion, HEK cells were repetitively stimulated with carbachol to assess the effectiveness of Ca^2+^ recycling to the ER after IP_3_-evoked Ca^2+^ release. Blocking lysosomal Ca^2+^ uptake with bafilomycin A_1_ increased the amplitude of each carbachol-evoked Ca^2+^ signal without affecting the rate of Ca^2+^ recycling to the ER. This suggests that Ca^2+^ accumulated by lysosomes is rapidly returned to the ER. We conclude that lysosomes rapidly, reversibly and selectively accumulate the Ca^2+^ released by IP_3_ receptors residing within distinct Ca^2+^ stores, but not the Ca^2+^ entering cells via receptor-regulated, store-operated Ca^2+^ entry pathways.

## Introduction

Ca^2+^ is a ubiquitous intracellular messenger [1], [2]. The intracellular free Ca^2+^ concentration ([Ca^2+^]_i_) is determined by Ca^2+^ transport across biological membranes and by high concentrations of cytosolic Ca^2+^ buffers [3]. Acute regulation of the Ca^2+^ signals that regulate most cellular activities is achieved by regulating Ca^2+^ transport, most often by controlling the opening of Ca^2+^-permeable channels within the plasma membrane or endoplasmic reticulum (ER) [1], [4]. The receptors for inositol 1,4,5-trisphosphate (IP_3_Rs) are the most prominent of the intracellular Ca^2+^ channels [5], [6]. The large conductance of IP_3_Rs and their regulation by both IP_3_ and Ca^2+^ allows them to release Ca^2+^ rapidly from the ER in response to the many receptors that stimulate phospholipase C (PLC), and then to mediate regenerative propagation of the cytosolic Ca^2+^ signals [7].

The ER is unique among intracellular organelles in the extent to which it forms intimate associations with other membranes [8], [9], [10] including mitochondria [11], the nucleus [12], lysosomes [13], [14] and the plasma membrane [15], [16]. It is becoming increasingly clear that these dynamic interactions between membranes play important roles in both shaping and decoding the Ca^2+^ signals evoked by physiological stimuli. Furthermore, rapid gating of the Ca^2+^ channels that initiate most Ca^2+^ signals and slow diffusion of Ca^2+^ within the cytosol allow local Ca^2+^-mediated communication between closely apposed membranes. The mitochondrial uniporter (MCU) [17], [18], for example, can rapidly sequester Ca^2+^ released by IP_3_Rs when mitochondria are locally exposed to high [Ca^2+^]_i_ near the mouths of open IP_3_Rs [11], [19], [20]. This both modulates IP_3_-evoked Ca^2+^ signals and regulates mitochondrial behaviour. Close apposition of STIM1 in ER membranes to Orai channels in the plasma membrane underlies regulation of the store-operated Ca^2+^ entry (SOCE) that almost invariably follows depletion of intracellular Ca^2+^ stores by IP_3_ [16]. More recently, lysosomes have also been suggested to contribute to regulation of [Ca^2+^]_i_ [13], [14], [21]. A variety of Ca^2+^-permeable channels expressed within lysosomal membranes, including two-pore channels (TPCs) [22], TRPML1 [23] and P2X4 receptors [24] have been proposed to mediate Ca^2+^ release in response to such stimuli as nicotinic acid adenine dinucleotide phosphate (NAADP) [22], [25], mTOR [26], phosphatidylinositol 3,5-bisphosphate [26], [27] and luminal ATP [24]. Again there is evidence of interactions with the ER, because NAADP-evoked Ca^2+^ release from lysosomes can be amplified by Ca^2+^ release from the ER mediated by Ca^2+^-activation of either IP_3_Rs or ryanodine receptors [28], [29].

**Figure 1:**
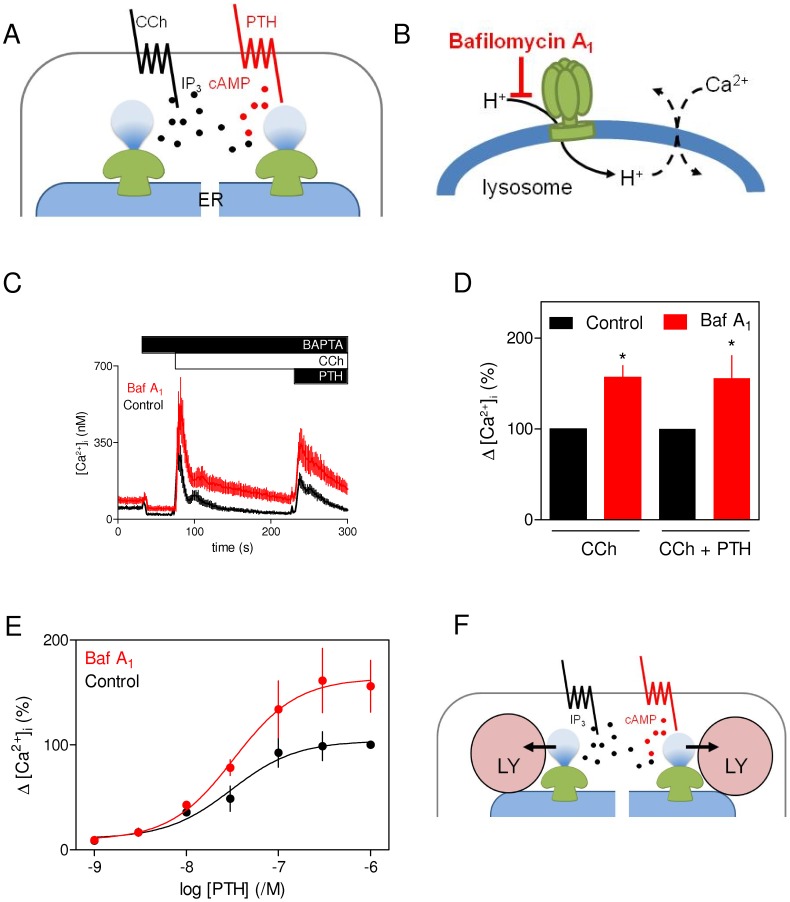
Lysosomes accumulate Ca^2+^ released from intracellular stores by IP_3_ alone or IP_3_ with cAMP. (A) CCh stimulates M_3_ muscarinic receptors leading to activation of PLC and IP_3_-evoked Ca^2+^ release from the ER. PTH, via type 1 PTH receptors, stimulates adenylyl cyclase. Cyclic AMP sensitizes IP_3_Rs to IP_3_ and thereby potentiates the Ca^2+^ release evoked by CCh. We suggest that cAMP is delivered to IP_3_Rs at high concentrations within signalling junctions [34] and that the IP_3_Rs that respond to CCh alone are activated by locally delivered IP_3_ [31]. This local signalling allows CCh alone and CCh in combination with PTH to release Ca^2+^ from different stores [31]. (B) Bafilomycin A_1_ (Baf A_1_) inhibits the V-ATPase that mediates H^+^ accumulation by lysosomes, and thereby prevents lysosomal Ca^2+^ uptake. The latter may be mediated by H^+^-Ca^2+^ exchange. (C) Populations of HEK-PR1 cells were stimulated with CCh (1 mM) and then PTH (1 µM) with or without prior treatment with bafilomycin A_1_ (1 µM, 1 h). BAPTA (10 mM) was added as shown to chelate extracellular Ca^2+^. Results are means ± S.E. from 3 wells from one experiment, typical of 3 similar experiments. (D) Summary results show effects of bafilomycin A_1_ on the amplitudes of the peak Ca^2+^ signals evoked by addition of CCh, or PTH after CCh. Results (as percentages of the responses without bafilomycin A_1_) are means ± S.E. from 3 independent experiments. (E) Experiments similar to those in C, show the effects of bafilomycin A_1_ on the concentration-dependent effects of PTH on CCh-evoked Ca^2+^ signals. Results are means ± S.E. from 3 independent experiments. (F) The results suggest that lysosomes (LY) accumulate Ca^2+^ released via IP_3_Rs activated by IP_3_ alone or IP_3_ with cAMP.

The mechanisms responsible for Ca^2+^ uptake into lysosomes are not known, although they require the pH gradient established across lysosomal membranes by the V-ATPase that pumps H^+^ into the lumen of lysosomes [14]. We [30] and others [28] recently provided evidence that lysosomes can also shape the Ca^2+^ signals evoked by IP_3_-evoked Ca^2+^ release from the ER. In our analysis, we demonstrated that dynamic lysosomes are associated with ER and that they selectively accumulate Ca^2+^ released by IP_3_Rs. But lysosomes do not sequester Ca^2+^ entering the cell via SOCE activated pharmacologically by inhibition of the SR/ER Ca^2+^-ATPase (SERCA) or by buffering of ER luminal Ca^2+^ [30]. Collectively, these observations suggest that lysosomes, like mitochondria [11], dynamically and intimately associate with ER. These associations contribute to both shaping IP_3_-evoked Ca^2+^ signals and to providing lysosomes with Ca^2+^ that might regulate their behaviour [30]. Here, we address three further questions relating to the interaction between lysosomes and IP_3_-evoked Ca^2+^ signals. First, we have argued that receptors, like the endogenous M_3_ muscarinic receptors of human embryonic kidney (HEK) cells, locally deliver IP_3_ to IP_3_Rs within signalling junctions, whereas different ‘extra-junctional’ IP_3_Rs release Ca^2+^ from distinct Ca^2+^ stores in response to lower concentrations of IP_3_ when their sensitivity is increased by cAMP [31] (Figure 1A). Do lysosomes sequester Ca^2+^ released from each of these IP_3_-sensitive Ca^2+^ stores? Second, does the SOCE evoked by physiological stimuli (rather than thapsigargin) direct Ca^2+^ to lysosomes? The answer to this question is important because it addresses whether a significant fraction of the Ca^2+^ entering cells via SOCE then passes through the ER and IP_3_Rs before re-entering the cytosol [32], [33]. Finally, and most importantly, are lysosomes ‘dead-end’ compartments for Ca^2+^, or is the Ca^2+^ they accumulate rapidly recycled to sustain refilling of ER Ca^2+^ stores?

## Results

### Disruption of lysosomal Ca^2+^ uptake exaggerates the Ca^2+^ signals evoked by Ca^2+^ release from distinct IP_3_-sensitive stores

Stimulation of the endogenous muscarinic M_3_ receptors of HEK cells with carbachol (CCh) activates PLC. The IP_3_ produced then evokes Ca^2+^ release from intracellular stores via IP_3_Rs (Figure 1A) [34]. Receptors that stimulate adenylyl cyclase, including heterologously expressed type 1 PTH receptors, potentiate the Ca^2+^ signals evoked by CCh [34]. This potentiation is entirely mediated by cAMP, which directly sensitizes IP_3_Rs to IP_3_ [34]. Previous work established that high concentrations of cAMP are delivered directly to IP_3_Rs from adenylyl cyclase within cAMP signalling junctions [34]. We recently presented evidence suggesting that the Ca^2+^ signals evoked by CCh alone result from local delivery of IP_3_ to IP_3_Rs that are probably closely associated with PLC [31]. We propose that this spatially organized delivery of diffusible messengers allows CCh alone to evoke Ca^2+^ release via IP_3_Rs from different Ca^2+^ stores to those that are released by CCh in combination with PTH (Figure 1A) [31].

A previous analysis of CCh-evoked Ca^2+^ signals in HEK cells demonstrated that lysosomes selectively accumulate the Ca^2+^ released from intracellular stores by CCh [30]. In light of evidence that CCh alone and CCh with PTH evoke Ca^2+^ release from different stores (Figure 1A) [31], we now assess whether the latter response is also modulated by lysosomal Ca^2+^ uptake. For these analyses, we used bafilomycin A_1_ selectively to inhibit H^+^ uptake by lysosomes (Figure 1B) [35] and thereby to prevent them from sequestering Ca^2+^. Previous work established that bafilomycin A_1_ is the most convenient way of disrupting lysosomal Ca^2+^ uptake, but other means of perturbing lysosomal function using GPN to perforate lysosomal membranes or vacuolin to affect the morphology and distribution of lysosomes had similar effects on CCh-evoked Ca^2+^ signals [30].

Pre-incubation of HEK cells stably expressing human type 1 PTH receptor (HEK-PR1 cells) with bafilomycin A_1_ caused the increase in [Ca^2+^]_i_ evoked by a maximally effective concentration of CCh in Ca^2+^-free HBS to increase by 1.5±0.2-fold (Figure 1C) [30]. PTH alone (1 µM) had no significant effect on [Ca^2+^]_i_ in HEK-PR1 cells (data not shown) [34], but it potentiated the Ca^2+^ signals evoked by CCh (Figure 1C). The increase in [Ca^2+^]_i_ evoked by addition of PTH in the continued presence of CCh was increased by 1.6±0.2-fold after pre-incubation with bafilomycin A_1_ (Figures 1C and 1D). The sensitivity to PTH was unaffected by bafilomycin A_1_: the pEC_50_ was 7.2±0.4 and 7.5±0.1 for control and bafilomycin A_1_-treated cells, respectively (where pEC_50_ is the -log of the half-maximally effective concentration) (Figure 1E). In these experiments, cells were first stimulated with CCh and then with PTH in the continued presence of CCh (Figure 1C). The similar effects of bafilomycin A_1_ on the first and second responses (Figure 1D) suggest that the capacity of lysosomes to sequester Ca^2+^ was unaffected by having accumulated Ca^2+^ during the first response to CCh. These results demonstrate that the Ca^2+^ signals resulting from Ca^2+^ release from two distinct IP_3_-sensitive Ca^2+^ stores are similarly affected by disruption of lysosomal Ca^2+^ uptake (Figure 1F).

### Attenuation of IP_3_-evoked Ca^2+^ signals by lysosomes does not require NAADP-activated channels

**Figure 2:**
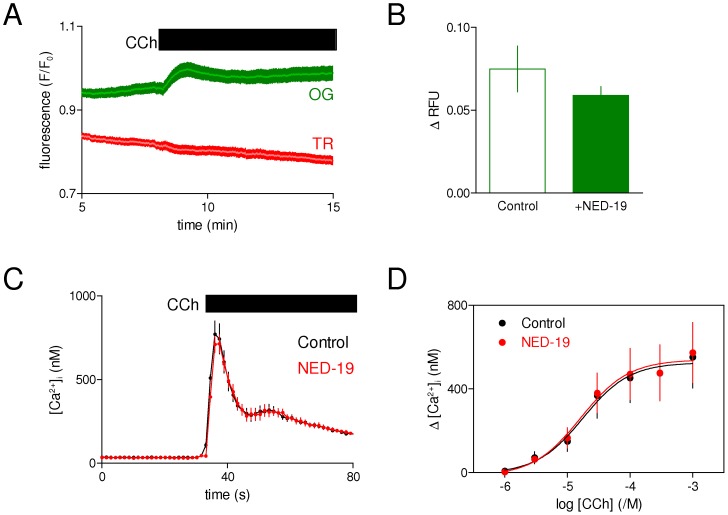
NAADP does not contribute to the effects of lysosomes on carbachol-evoked Ca^2+^ release in HEK cells. (A) HEK cells loaded with dextran-conjugates of Oregon Green (OG, pH-sensitive probe) and Texas Red (TR, inert marker) were stimulated with CCh (1 mM). Results (means ± S.E. from 27 ROI on a single coverslip, representative of at least 3 independent experiments) show that CCh causes the pH of the lysosome lumen to increase. Addition of HBS did not affect OG or TR fluorescence [30]. (B) Similar experiments with and without NED-19 (10 µM, 1 h) show that it has no significant effect on the peak increase in lysosomal pH evoked by CCh. Results (means ± S.E. from 7 experiments) show the peak change in OG fluorescence (ΔRFU, relative fluorescence units). (C) [Ca^2+^]_i_ was recorded from HEK cells stimulated with CCh (1 mM) alone or with NED-19 (10 µM, 1 h). Results show means ± S.E. from 3 wells in one experiment, typical of 3 experiments. (D) Summary results show the lack of effect of NED-19 on the peak Ca^2+^ signals evoked by CCh. Results are means ± S.E. from 3 experiments.

In sea urchin eggs, IP_3_-evoked Ca^2+^ release triggers a rapid increase in the luminal pH of lysosomes [28]. We observed a similar response in CCh-stimulated HEK cells [30] (Figure 2A) and attributed it to an exchange of lysosomal H^+^ for cytosolic Ca^2+^ [30]. Morgan *et al*., however, suggest a different interpretation for their results. They argue that Ca^2+^ release from sea urchin lysosomes increases lysosomal pH, and that IP_3_-evoked Ca^2+^ release elicits the same response by locally stimulating formation of NAADP and perhaps also by a direct effect of cytosolic Ca^2+^ on NAADP-evoked Ca^2+^ release [28]. It is unlikely that such interactions contribute to the effects of lysosomes on IP_3_-evoked Ca^2+^ signals in HEK cells. Firstly, active lysosomes attenuate IP_3_-evoked Ca^2+^ signals in HEK cells (Figure 1), while they are proposed to amplify them in sea urchin eggs [28]. Secondly, NED-19, an antagonist of NAADP [36], had no effect on the alkalinization of lysosomal pH during stimulation of HEK cells with CCh (Figure 2B). Furthermore, NED-19 did not affect the time course of the Ca^2+^ signals evoked by a maximally effective concentration of CCh (Figure 2C) or the peak response to any concentration of CCh (Figure 2D).

In both sea urchin eggs and HEK cells, ER and lysosomes are closely apposed [28], [30], but the nature of the Ca^2+^-mediated ‘chatter’ between IP_3_Rs and lysosomes seems to be configured differently. In sea urchin eggs, IP_3_-evoked Ca^2+^ release appears to be amplified by NAADP-evoked Ca^2+^ release from lysosomes [28], while in HEK cells lysosomes rapidly sequester the Ca^2+^ released by IP_3_Rs (Figure 1).

### Ca^2+^ signals resulting from carbachol-evoked Ca^2+^ entry are not affected by lysosomes

**Figure 3:**
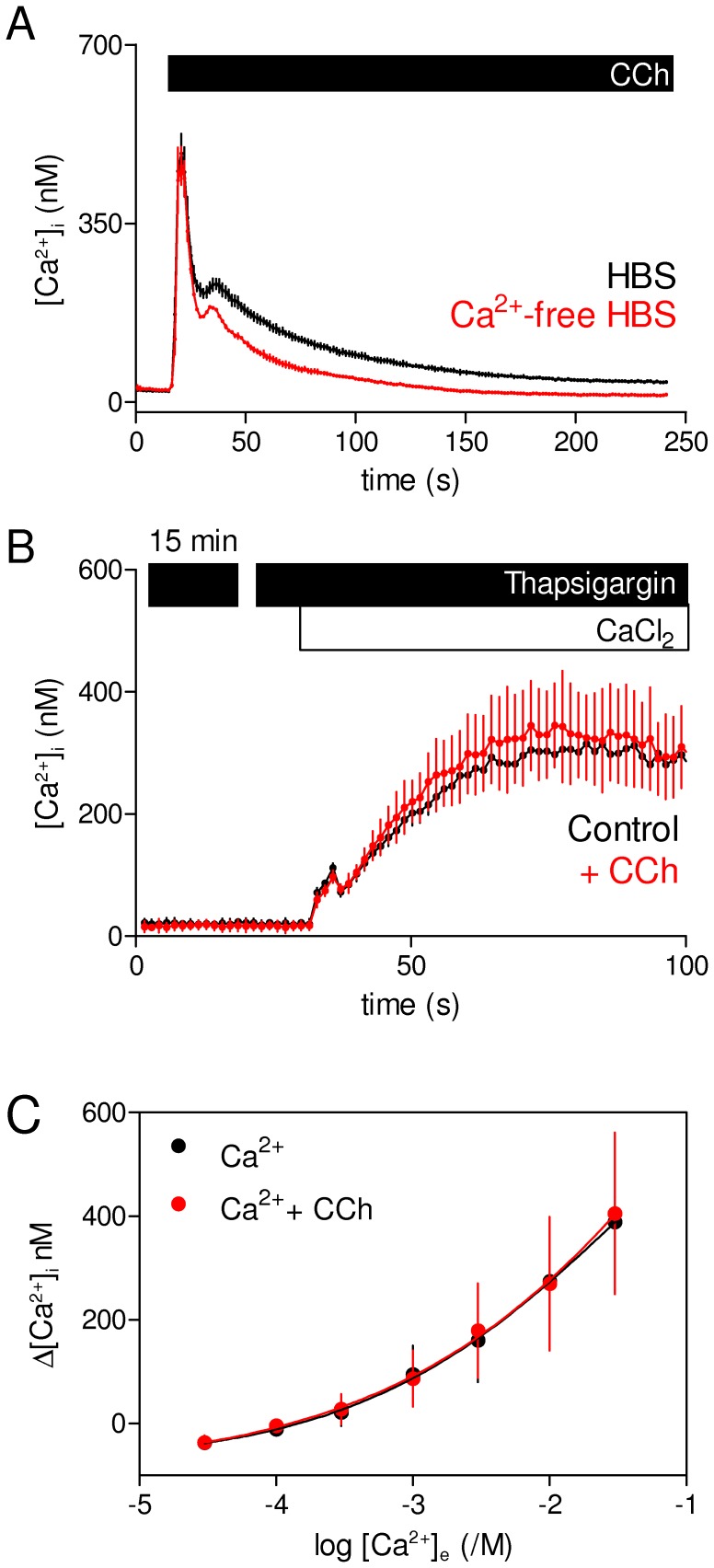
Carbachol evokes store-operated Ca^2+^ entry in HEK-PR1 cells. (A) Typical responses of a population of HEK-PR1 cells stimulated with CCh (1 mM) in HBS with or without extracellular Ca^2+^. For the latter BAPTA (10 mM) was added with CCh. (B) HEK-PR1 cells were incubated with thapsigargin (1 µM, 15 min) in nominally Ca^2+^-free HBS before restoration of extracellular Ca^2+^ (30 mM) alone or with CCh (1 mM). Results (A and B) show means ± S.E. from 3 replicates of a single experiment, representative of at least 3 similar experiments. (C) Similar experiments show the peak amplitude of the Ca^2+^ signal evoked by restoration to thaspsigargin-treated cells of the indicated concentrations of extracellular Ca^2+^ ([Ca^2+^]_e_) alone or with CCh (1 mM). Results are means ± S.E. from 3 independent experiments.

CCh evokes both IP_3_-mediated release of Ca^2+^ from intracellular stores (Figure 1A and 1C) and Ca^2+^ entry across the plasma membrane (Figure 3A). In most cells, including HEK cells (Figures 3B and 3C) [30], [37], depletion of intracellular Ca^2+^ stores activates SOCE [38]. But receptors that activate PLC can also stimulate additional Ca^2+^ entry pathways, including those that are regulated by arachidonic acid [39], [40]. Whether such Ca^2+^ entry pathways contribute to CCh-evoked Ca^2+^ entry in HEK cells is controversial [37], [41], [42]. In HEK-PR1 cells, CCh affected neither the time course of the Ca^2+^ signals evoked by restoration of extracellular Ca^2+^ to thapsigargin-treated cells, nor the amplitude of these signals when the extracellular Ca^2+^ concentration was varied (Figures 3B and 3C). These results suggest that the Ca^2+^ entry evoked by CCh in HEK-PR1 cells is mediated by SOCE.

**Figure 4:**
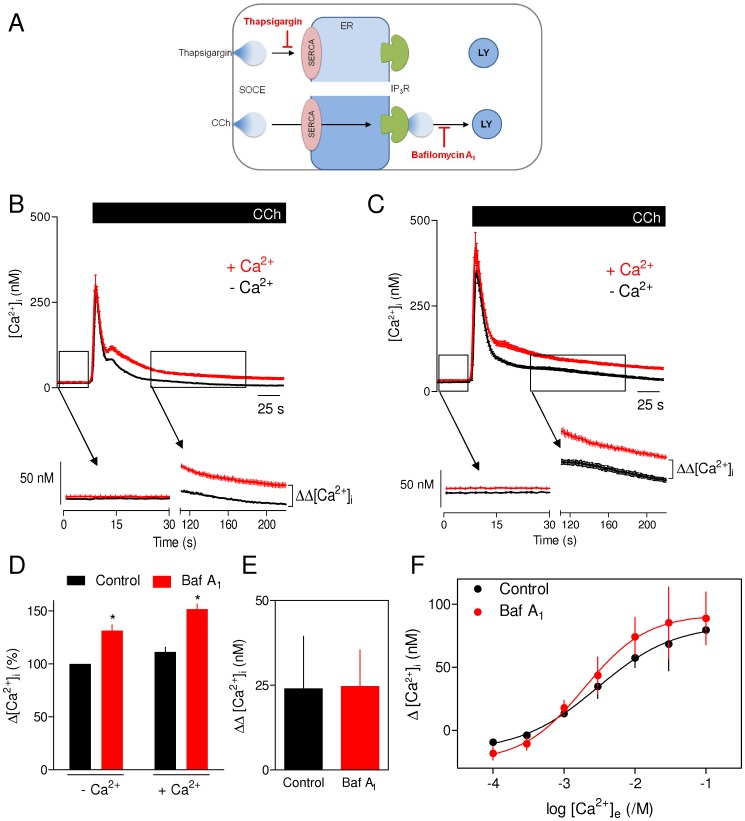
Lysosomes do not accumulate Ca^2+^ entering cells via store-operated Ca^2+^ entry evoked by carbachol. (A) Ca^2+^ entering cells via SOCE evoked by CCh may pass through the ER and then re-enter the cells via IP_3_Rs from which some Ca^2+^ might then be accumulated by lysosomes (LY). That route is impossible when the SERCA is inhibited by thapsigargin. (B, C) Cells were stimulated with CCh (1 mM) in normal or Ca^2+^-free HBS alone (B) or with bafilomycin A_1_ (1 µM, 1 h) (C). The enlargements beneath the panels illustrate how the component of the Ca^2+^ signal attributable to Ca^2+^ entry (ΔΔ[Ca^2+^]_i_) was calculated. Results show means ± S.E. from 6 replicates from a single experiment, typical of 4 similar experiments. (D) Peak increases in [Ca^2+^]_i_ evoked by CCh in normal or Ca^2+^-free HBS, with and without bafilomycin A_1_-treatment. Results (percentages of the responses to CCh alone in Ca^2+^-free HBS) are means ± S.E. from 4 experiments. *p <0.05, paired Students's t-test using the raw data. (E) Similar analysis (means ± S.E., n  =  4) shows ΔΔ[Ca^2+^]_i_ recorded 2 min after CCh addition. (F) Cells were stimulated with CCh (1 mM, 15 min) in nominally Ca^2+^-free HBS with or without bafilomycin A_1_ (1 µM, 1 h) before restoration of the indicated concentrations of extracellular Ca^2+^. Results (means ± S.E., n  =  4) show the sustained increase in [Ca^2+^]_i_.

Our previous analysis established that lysosomes selectively accumulate Ca^2+^ released from the ER, but not Ca^2+^ entering cells via SOCE evoked by thapsigargin [30]. It is not known whether lysosomes affect SOCE evoked by CCh. The question is important because Ca^2+^ entering the cell via SOCE can locally regulate specific intracellular events [43], [44], but it is unclear whether it can also pass through the ER and so re-enter the cytosol via IP_3_Rs [32], [33]. The latter route is impossible when the SR/ER Ca^2+^-ATPase (SERCA) is inhibited by thapsigargin (Figure 4A). We therefore considered the possibility that CCh-evoked SOCE might be modulated by lysosomal Ca^2+^ uptake systems if a significant fraction of the Ca^2+^ entering by SOCE passed through the ER via SERCA and IP_3_Rs (Figure 4A). Evidence that CCh-evoked Ca^2+^ entry in HEK-PR1 cells is mediated by SOCE (Figure 3) [37] allows this issue to be addressed

The results shown in Figure 4B establish that the increase in [Ca^2+^]_i_ resulting from CCh-evoked release of intracellular Ca^2+^ stores is complete within 2 min, whereas the small Ca^2+^ signal mediated by SOCE persists for much longer. We therefore analysed the increases in [Ca^2+^]_i_ (Δ[Ca^2+^]_i_) detected 2 min after CCh addition in the absence and presence of extracellular Ca^2+^ to assess the effects of bafilomycin A_1_ on CCh-evoked SOCE. The difference between these values (ΔΔ[Ca^2+^]_i_  = Δ[Ca^2+^]_i_ with extracellular Ca^2+^- Δ[Ca^2+^]_i_ without extracellular Ca^2+^) reports the magnitude of the CCh-evoked SOCE (Figures 4B and 4C).

Bafilomycin A_1_ potentiated the initial peak increase in [Ca^2+^]_i_ evoked by CCh in both the absence and presence of extracellular Ca^2+^ by 1.3±0.07 and 1.3±0.04-fold, respectively (Figures 4B–4D). This is consistent with sequestration by lysosomes of Ca^2+^ released by IP_3_Rs [30]. Because bafilomycin A_1_ slows the recovery of [Ca^2+^]_i_ during IP_3_-evoked Ca^2+^ release [30], [Ca^2+^]_i_ was still higher in bafilomycin A_1_-treated relative to control cells after a 2-min exposure to CCh in Ca^2+^-free HBS (compare the black traces in Figures 4B and 4C). More importantly, however, bafilomycin A_1_ had no effect on ΔΔ[Ca^2+^]_i_, which was 24±8 nM and 25±5 nM for control and bafilomycin A_1_-treated cells, respectively (Figure 4E). These results suggest that CCh-evoked SOCE is insensitive to bafilomycin A_1_.

The amplitudes of the sustained Ca^2+^ signals evoked by CCh in normal HBS are small relative to those resulting from IP_3_-evoked Ca^2+^ release (Figures 4B and 4C). We therefore examined the effects of bafilomycin A_1_ on CCh-evoked Ca^2+^ entry under conditions that temporally separated Ca^2+^ release from Ca^2+^ entry. We also used higher concentrations of extracellular Ca^2+^ to exaggerate the Ca^2+^ entry signals. Cells were treated with CCh in nominally Ca^2+^-free HBS for 15 min to deplete the ER and activate SOCE. Different extracellular Ca^2+^ concentrations were then restored in the continued presence of CCh. The results demonstrate that bafilomycin A_1_ has no effect on the sustained phase of the resulting increase in [Ca^2+^]_i_ at any extracellular Ca^2+^ concentration (Figure 4F). These results suggest that CCh-evoked SOCE, like that evoked by thapsigargin [30], is insensitive to inhibition of lysosomal Ca^2+^ uptake.

### Lysosomes recycle the Ca^2+^ accumulated after stimulation of IP_3_ receptors

The results so far demonstrate that in HEK cells stimulated with CCh, lysosomes selectively sequester Ca^2+^ released via IP_3_Rs, but not Ca^2+^ entering the cell via SOCE (Figures 1 and 4). We next assessed whether Ca^2+^ accumulated by lysosomes remains trapped within them or gets rapidly recycled to the ER via the cytosol (Figure S1A).

**Figure 5:**
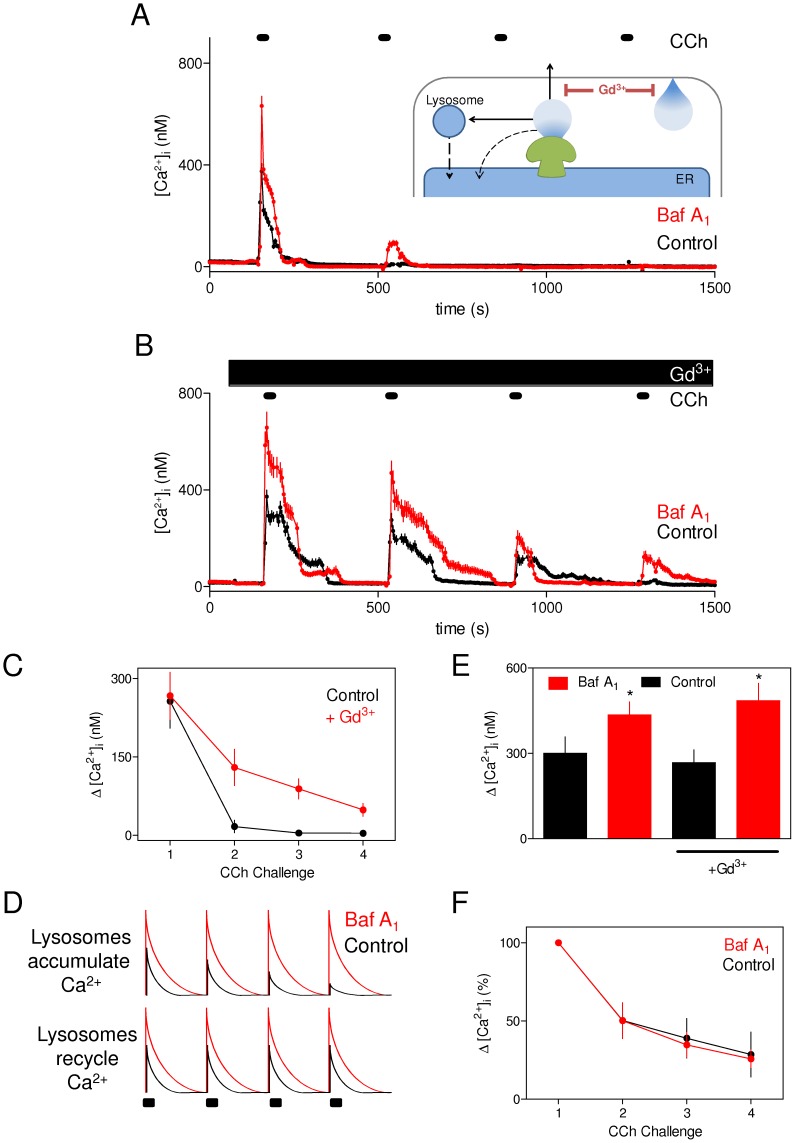
Lysosomes rapidly recycle the Ca^2+^ sequestered after IP_3_-evoked Ca^2+^ release. (A, B) HEK cells were repetitively stimulated with CCh (1 mM, 30 s) alone or with bafilomycin A_1_ (1 µM, 1 h) in nominally Ca^2+^-free HBS without (A) or with Gd^3+^ (1 mM) (B). Results show means ± S.E. for ≥ 45 cells from a single experiment, typical of at least 3 similar experiments. The inset to panel A shows how a high concentration of Gd^3+^ (1 mM) effectively insulates the cell from exchanging Ca^2+^ with the extracellular environment by blocking Ca^2+^ entry and extrusion [37]. Under these conditions, repetitive responses to CCh are entirely dependent on recycling of intracellular Ca^2+^ (dashed lines). (C) Summary results show effects of Gd^3+^ on the peak increase in [Ca^2+^]_i_ evoked by each challenge with CCh in the absence of bafilomycin A_1_. (D) Predicted effects of bafilomycin A_1_ on the Ca^2+^ signals evoked by repetitive CCh challenges of Gd^3+^-insulated cells. The predicted results represent an idealized situation in which Gd^3+^ entirely insulates the cell from Ca^2+^ exchanges with the extracellular environment (in practise the insulation is incomplete), and then shows the results predicted for situations where lysosomes either accumulate (upper panel) or entirely recycle (lower panel) the sequestered Ca^2+^ (see Figure S1A). (E) Peak increases in [Ca^2+^]_i_ evoked by the first CCh challenge under the conditions shown. *p <0.05, paired Students's t-test. (F) Effects of bafilomycin A_1_ on the peak increases in [Ca^2+^]_i_ evoked by successive CCh challenges in nominally Ca^2+^-free HBS containing 1 mM Gd^3+^. Results are normalized to the first CCh challenge for each condition (the raw data and the results obtained in the absence of Gd^3+^ are shown in Figure S1B and S1C). Results (C, E and F) are means ± S.E. from at least 4 independent experiments.

To address this issue, HEK cells were stimulated with CCh under conditions (1 mM GdCl_3_ in the extracellular medium) that inhibit both Ca^2+^ extrusion across the plasma membrane and Ca^2+^ entry [37] (Figure 5A inset). Comparison of the black traces in Figures 5A and 5B, where HEK cells in nominally Ca^2+^-free HBS were repeatedly stimulated with brief pulses of a maximally effective concentration of CCh (1 mM), demonstrates that the approach is effective, albeit without fully preventing loss of Ca^2+^ from stimulated cells. The incomplete inhibition of Ca^2+^ loss by Gd^3+^ contrasts with a previous analysis of HEK cells where CCh-evoked Ca^2+^ oscillations persisted for many minutes with undiminished amplitude in Ca^2+^-free medium supplemented with 1 mM Gd^3+^ [37]. The different results probably result from the much higher concentration of CCh used in our experiments (1 mM) relative to that used to evoke Ca^2+^ oscillations (1–5 µM) [37]. In Ca^2+^-free HBS, cells responded robustly to the first CCh challenge, but not to subsequent challenges (Figure 5A). In the same HBS supplemented with Gd^3+^, even the fourth challenge with CCh evoked a detectable increase in [Ca^2+^]_i_ (Figures 5B and 5C). These results confirm that a substantial fraction of the Ca^2+^ released from intracellular stores by IP_3_ is normally extruded from the cell. That Ca^2+^ would normally be replenished by SOCE, but in the absence of extracellular Ca^2+^ the stores are unable to refill. A high concentration of Gd^3+^, by inhibiting Ca^2+^ exchanges across the plasma membrane (both influx and efflux), allows Ca^2+^ to be recycled within the cell and thereby allows the ER to respond to repeated CCh challenges (Figures 5B and 5C).

Some of the Ca^2+^ released by IP_3_Rs is sequestered by lysosomes (Figure 1). If that sequestered Ca^2+^ were only very slowly recycled to the ER (i.e. more slowly than the 5-min interval between the CCh challenges shown in Figure 5), the effect of lysosomes would be analogous to Ca^2+^ extrusion across the plasma membrane (Figure S1A). The lysosomes would then effectively remove Ca^2+^ from the recycling pool, just as Ca^2+^ extrusion across the plasma membrane in Ca^2+^-free medium effectively depletes the pool of Ca^2+^ available for signalling. The amplitude of the Ca^2+^ signals evoked by repetitive pulses of CCh under ‘Gd^3+^-insulating’ conditions would then be expected to decay more quickly when lysosomes are active because with each Ca^2+^ spike lysosomes would effectively remove some Ca^2+^ from the recycling pool. A cartoon representation of the predicted effects of bafilomycin A_1_ on the Ca^2+^ signals evoked by repetitive CCh challenges is shown in idealized form in Figure 5D, which assumes that Gd^3+^ is entirely effective in preventing Ca^2+^ fluxes across the plasma membrane. Bafilomycin A_1_ is predicted to have no effect on the run-down of CCh-evoked Ca^2+^ signals if Ca^2+^ is rapidly recycled from lysosomes, and to slow the run-down if lysosomes normally retain the sequestered Ca^2+^ and so remove it from the signalling pool (Figure 5D). We tested these predictions by measuring the effects of bafilomycin A_1_ on the responses to repeated brief (30 s) challenges with CCh in nominally Ca^2+^-free HBS supplemented with 1 mM Gd^3+^ (Figures 5A and 5B).

As expected, bafilomycin A_1_ potentiated the increase in [Ca^2+^]_i_ evoked by CCh in both the absence and presence of Gd^3+^ (1.8±0.4 and 1.7±0.2-fold increase, respectively) (Figure 5E). It is, however, noteworthy that the peak amplitude of the CCh-evoked Ca^2+^ signal was unaffected by Gd^3+^ (Figures 5A–5C and 5E). This suggests that Ca^2+^ sequestration by lysosomes is fast enough to attenuate the initial IP_3_-evoked Ca^2+^ release signal, while extrusion of Ca^2+^ across the plasma membrane is either too slow or too far removed from the site of Ca^2+^ release to detectably affect the initial rise in [Ca^2+^]_i_.

Neither bafilomycin A_1_ nor Gd^3+^ affected the number of cells responding to the initial CCh challenge (Table 1). However, responses to each successive CCh challenge were larger in the presence of bafilomycin A_1_ (Figures 5A and 5B). This confirms that each CCh challenge normally evokes a sequestration of Ca^2+^ by lysosomes. Despite the larger CCh-evoked Ca^2+^ signals in the presence of bafilomycin A_1_, the rate at which the peak amplitude of the Ca^2+^ signal declined with each successive CCh challenge was identical in control and bafilomycin A_1_-treated cells (Figure 5F, Figure S1B and S1C). These results suggest that lysosomes rapidly recycle the Ca^2+^ they accumulate during IP_3_-evoked Ca^2+^ release (lower panel in Figure 5D and Figure S1A).

**Table 1:** Neither Gd^3+^ nor bafilomycin A_1_ affects the number of cells that respond to carbachol.

	Control	+ Bafilomycin A_1_
Control	89 ± 6%	87 ± 9%
+ Gd^3+^	77 ± 8%	79 ± 10%

Single-cell analyses show the percentages of cells in which CCh (1 mM) evoked a detectable increase in [Ca^2+^]_i_ in cells treated with Gd^3+^ and/or bafilomycin A_1_ under exactly the conditions used for Figure 5. Results are from ≥ 3 independent experiments, with ∼70 cells analysed in each.

## Discussion

**Figure 6:**
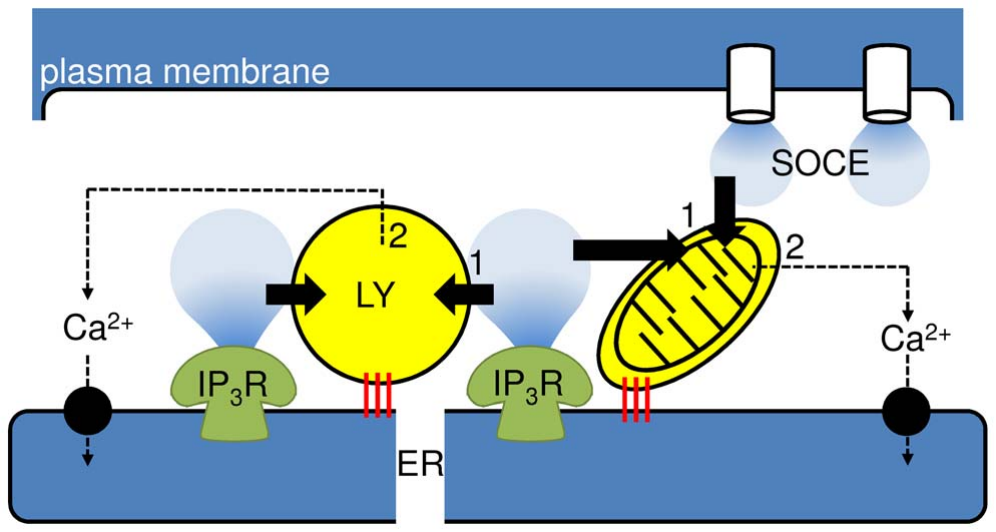
Associations of ER with other Ca^2+^-sequestering organelles allows selective and reversible modulation of cytosolic Ca^2+^ signals. Close association of lysosomes (LY) with ER [30], probably mediated by specific tethers (red) [46], allows them selectively to accumulate Ca^2+^ released by IP_3_Rs from distinct ER Ca^2+^ stores, but not Ca^2+^ entering the cell via SOCE. Mitochondria (right), depending on cell type, can selectively accumulate Ca^2+^ released from the ER, to which they are tethered, or entering the cell via SOCE [11]. For lysosomes, neither the Ca^2+^ uptake pathway (1) nor the efflux pathway (2) that rapidly recycles Ca^2+^ back to the ER via the cytosol have been identified. The equivalent pathways in mitochondria are the MCU (1) and Na^+^/Ca^2+^ or H^+^/Ca^2+^ exchangers (2) of the inner mitochondrial membrane. Rapid, reversible and selective ‘buffering’ of cytosolic Ca^2+^ signals by both lysosomes and mitochondria allows these organelles to both shape and decode stimulus-evoked Ca^2+^ signals.

We have shown that lysosomes sequester Ca^2+^ released from the ER [30]. The present work demonstrates that different IP_3_-sensitive Ca^2+^ stores within the compartmentalized ER of HEK cells [31] are each capable of directing the Ca^2+^ released by IP_3_Rs to lysosomal Ca^2+^ uptake systems (Figures 1 and 6). By contrast the Ca^2+^ signals evoked by SOCE, whether activated pharmacologically [30] or by endogenous receptors that stimulate PLC (Figure 4), are insensitive to inhibition of lysosomes. This is not due to the small amplitude of SOCE-mediated Ca^2+^ signals (Figures 3A and 4) because SOCE remains insensitive to inhibition of lysosomes when SOCE-evoked increases in global [Ca^2+^]_i_ are larger than those evoked by IP_3_Rs [30]. The insensitivity of CCh-evoked SOCE to inhibition of lysosomal Ca^2+^ uptake suggests two important conclusions. First, it reinforces our suggestion that lysosomes selectively sequester Ca^2+^ released by IP_3_Rs [30]. The intimacy of the relationship between ER and lysosomes is further supported by the different effects of inhibiting lysosomes (Figure 1) or Ca^2+^ extrusion across the plasma membrane (Figure 5C). Only the former increases the amplitude of the initial CCh-evoked increase in [Ca^2+^]_i_, suggesting that only lysosomes are both close enough to IP_3_Rs and accumulate Ca^2+^ fast enough to attenuate the initial response to IP_3_. Second, it suggests that during SOCE in HEK cells, there is probably no significant flux of Ca^2+^ from Orai channels into the ER and then back into the cytosol via IP_3_Rs (Figure 4A lower panel).

Our observations are consistent with evidence that lysosomes are both closely associated with ER [28], [30], [45], [46] and maintain their association as each organelle moves [30]. This relationship is reminiscent of that between ER and mitochondria [30], where tethering of the two organelles at mitochondria-associated membranes (MAMs) allows local exchange of Ca^2+^ and lipids [11], [47]. The mitochondrial uniporter (MCU) mediates Ca^2+^ uptake by mitochondria, whereas mitofusin 2 [48] and perhaps other proteins that may include IP_3_Rs [9], [11], contribute to formation of mitochondrion-ER junctions. For lysosomes, neither the Ca^2+^ uptake mechanism [14] nor the ER tethers are known, although both are important questions for future work. Tethering of ER to the vacuole in yeast (analogous to the acidic organelles of higher eukaryotes) is mediated by interaction of proteins anchored to ER (NVJ1) and vacuolar membranes (Vac8). These then recruit Osh1, an oxysterol-binding protein-related protein (ORP) and a lipid-synthesizing enzyme (Tcs13) [9]. Interactions between ORPs [49] or other lipid-binding proteins like STARD3 (steroidogenic acute regulatory protein domain 3) [46], and the ER protein, VAP (VAMP-related proteins), may contribute to assembly of ER-lysosome junctions in higher eukaryotes [50]. We speculate that these, or additional tethering proteins, may maintain the close association between lysosomes and ER required to allow lysosomes to accumulate Ca^2+^ selectively and rapidly in response to its release by IP_3_Rs (Figure 6).

Mitochondrial Ca^2+^ uptake plays an important role in buffering cytosolic Ca^2+^ signals [11]. The capacity of mitochondria to modulate [Ca^2+^]_i_ is abrogated when mitochondrial Ca^2+^ efflux is inhibited [51], [52]. Furthermore, temporal changes of [Ca^2+^] within mitochondria faithfully track even quite rapid oscillations in [Ca^2+^]_i_ [53]. These observations suggest that mitochondria can rapidly recycle at least some of the Ca^2+^ they accumulate from the cytosol, and that rapid shuttling of Ca^2+^ between the ER and mitochondria contributes to both cytosolic Ca^2+^ oscillations [54] and mitochondrial activity [53]. We suggest a similar situation for lysosomes (Figure 6), although neither the Ca^2+^ uptake nor efflux pathways are resolved for lysosomes. It is clear from experiments where cells were first stimulated with CCh and then with CCh and PTH (Figure 1) that the ability of lysosomes to sequester Ca^2+^ is unaffected by prior Ca^2+^ sequestration. This suggests that lysosomes have a considerable capacity to accumulate Ca^2+^, or that having sequestered Ca^2+^ they can rapidly recycle it, via the cytosol, to other organelles. We used Gd^3+^ to ‘insulate’ cells from Ca^2+^ exchanges with the extracellular environment and so force them into relying on recycling of intracellular Ca^2+^ pools to generate increases in [Ca^2+^]_i_ [37]. Under these conditions, we demonstrated that successive responses to CCh were each exaggerated by inhibition of lysosomes, but the rate at which Ca^2+^ was lost from the recycling pool of Ca^2+^ was unaffected (Figure 5 and Figure S1). These results suggest that lysosomes rapidly recycle the Ca^2+^ that they accumulate (Figure 6). This conclusion is consistent with evidence that inhibition of lysosomes increases the amplitude, but decreases the frequency, of the Ca^2+^ spikes evoked by low concentrations of CCh [30]. The latter reflecting the slower, but still effective, recycling of Ca^2+^ from the cytosol to ER when lysosomes are active.

We conclude that lysosomes rapidly, reversibly and selectively accumulate Ca^2+^ released by IP_3_Rs, even when the IP_3_Rs reside in distinct Ca^2+^ stores, but they are unable to accumulate Ca^2+^ entering cells via SOCE (Figures 1–4). The behaviour of lysosomes provides a striking analogy with mitochondria [30]. Both organelles rapidly accumulate Ca^2+^ from microdomains surrounding specific Ca^2+^ channels and thereby shape cytosolic Ca^2+^ signals [11], [30] (Figures 1, 4 and 5), and both are capable of rapidly recycling the accumulated Ca^2+^ [51], [52], [53], [54] (Figure 5). Finally, for both organelles the increase in luminal [Ca^2+^] regulates their activity: enzyme activity, apoptosis and motility for mitochondria [11], and endo-lysosomal trafficking [55] and perhaps ion channel activity [56] for lysosomes (Figure 6).

## Materials and Methods

### Materials

Dulbecco's modified Eagle's/Ham's F-12 (DMEM/F-12), fluo-4-AM, fura-2-AM, dextran-conjugates of Oregon Green (M_r_ = 10,000) and Texas Red (M_r_ = 10,000), and Ca^2+^ standard solutions were from Invitrogen (Paisley, U.K.). NED-19 was from Enzo Life Sciences (Exeter, U.K.). G418 was from Formedium (Norfolk, U.K.). Cell culture plastics and 96-well plates were from Greiner (Stonehouse, Gloucestershire, U.K.). Imaging dishes (35-mm diameter with a 7-mm No. 0 glass insert) were from MatTek Corporation (Ashland, MA, U.S.A.) or PAA Laboratories (Yeovil, U.K.). Carbamyl choline chloride (carbachol, CCh), DMSO, foetal bovine serum (FBS), poly-l-lysine, Pluronic F127 and Triton-X-100 were from Sigma-Aldrich (Poole, Dorset, U.K.). BAPTA (1,2-bis(o-aminophenoxy)ethane-N,N,N′,N′-tetraacetic acid) was from Molekula (Dorset, U.K.). Bafilomycin A_1_ was from AG scientific (San Diego, CA, U.S.A.). Parathyroid hormone (PTH, residues 1–34) was from Bachem (St. Helens, U.K.). Ionomycin was from Merck Eurolab (Nottingham, U.K.). Thapsigargin was from Alomone Labs (Jerusalem, Israel).

### Cell culture

HEK cells and HEK cells stably expressing human type 1 PTH receptors (HEK-PR1 cells) were cultured at 37°C in DMEM/F-12 medium with GlutaMAX-1, FBS (10%) and G418 (800 µg/ml for HEK-PR1 cells) in humidified air with 5% CO_2_. For experiments, cells were seeded into 96-well plates or onto 22-mm round glass coverslips coated with 0.01% (w/v) poly-l-lysine.

### Measurements of [Ca^2+^]_i_

[Ca^2+^]_i_ in populations of confluent cells loaded with fluo-4 was measured at intervals of 1.44 s using a fluorescence plate-reader as described previously [34]. Cells were incubated at 20°C in HEPES-buffered saline (HBS: NaCl 135 mM, KCl 5.9 mM, MgCl_2_ 1.2 mM, CaCl_2_ 1.5 mM, HEPES 11.6 mM and glucose 11.5 mM, pH 7.3). Ca^2+^ was omitted from nominally Ca^2+^-free HBS, and replaced by BAPTA (10 mM) in Ca^2+^-free HBS. Fluorescence (F) was calibrated to [Ca^2+^]_i_ from [Ca^2+^]_i_ = K_D_(F-F_min_)/(F_max_-F), where K_D_ is the dissociation constant of fluo-4 for Ca^2+^ (345 nM), F_min_ and F_max_ are the fluorescence signals recorded after treatment of parallel wells with Triton X-100 (0.1% v/v) in HBS supplemented with 10 mM BAPTA or 10 mM CaCl_2_, respectively. Concentration-effect relationships were fitted to Hill equations using non-linear curve-fitting (GraphPad Prism, version 5).

For single-cell imaging, confluent cultures of HEK cells on 22-mm round, poly-l-lysine-coated glass coverslips were loaded with fura-2-AM (2 µM, 1 h) supplemented with Pluronic F127 (0.02% v/v), washed and incubated for a further 1 h in HBS. Fluorescence, detected at>510 nm after alternating excitation at 340 and 380 nm, was detected using an Olympus IX71 inverted fluorescence microscope with a Luca EMCCD camera (Andor Technology, Belfast, U.K.). After correction for background fluorescence by addition of MnCl_2_ (10 mM) and ionomycin (1 µM) at the end of the experiment, fluorescence ratios (F_340_/F_380_) were calibrated to [Ca^2+^]_i_ using Ca^2+^ standard solutions [34]. Only cells that responded to the first stimulation with CCh (typically>80% of cells) were included in the analysis of Ca^2+^ signals evoked by successive CCh challenges (see Figure 5).

### Measurement of lysosomal pH

Almost confluent cultures of HEK cells grown on poly-l-lysine-coated, glass-bottomed dishes were incubated in culture medium with dextran-conjugates of Texas Red (TR, 0.1 mg/ml, an inert marker) and Oregon Green (OG, 0.1 mg/ml, a pH indicator) for 12 h at 37°C to allow uptake of the indicators by endocytosis. After a further incubation (4 h) without indicators, the cells were washed with HBS and fluorescence was recorded in HBS at 20°C using an Olympus IX81 microscope with a 60x/1.45 NA objective. Cells were illuminated with a mercury xenon lamp using alternating filter sets: U-MNIBA (Olympus, λ_ex_ 470–495 nm, λ_em_ 510–550 nm for OG) and LF561A (Semrock, λ_ex_ 550–570 nm, λ_em_ 580–630 nm for TR). Images were captured at 2-s intervals using an EMCCD camera (Andor iXon 897) and analyzed using Cell∧R software (Olympus, Milton Keynes, U.K.). Records were corrected for background fluorescence determined under identical conditions from cells without indicators. Fluorescence changes from defined regions of interest (ROI) are expressed as F/F_0_, where F_0_ and F denote the average fluorescence within the ROI at the start of the experiment (F_0_) and at each time point (F).

## Supporting Information

Figure S1 **Responses to repetitive challenges with carbachol reveal that Ca^2+^ rapidly recycles from lysosomes.** (A) A fraction of the Ca^2+^ released from the ER via IP_3_Rs is normally lost to the extracellular space as Ca^2+^ pumps in the plasma membrane (PM) extrude it from the cytosol. When Ca^2+^ is present in the extracellular medium, this loss is replenished by store-operated Ca^2+^ entry (SOCE). Removal of extracellular Ca^2+^ or blockade of SOCE by Gd^3+^ prevents this recycling of Ca^2+^. Lysosomes also sequester Ca^2+^ released by IP_3_Rs [30], but it is important to resolve whether that Ca^2+^ is also rapidly recycled via the cytosol to the ER. The experiments shown in Figure 5 address this issue. (B) The Ca^2+^ signals evoked by repetitive challenges with CCh (1 mM, 30 s) were recorded from HEK cells in Ca^2+^-free HBS with 1 mM Gd^3+^ (as shown in Figure 5B). The peak amplitudes of the Ca^2+^ signals are shown for control cells and cells treated with bafilomycin A_1_ (means ±S.E., n = 6). These raw data were used to produce Figure 5F. (C) Summary data (means ±S.E., n = 6) from experiments similar to those shown in (B), but in Ca^2+^-free HBS, show that in the absence of high concentrations of Gd^3+^, cells respond robustly to the first CCh challenge, but not to subsequent challenges.
(TIF)
