# Supplementary figures and images for: Rapid Recycling of Ca2+ between IP3-Sensitive Stores and Lysosomes
Source: PLoS One. 2014 Oct 22;9(10):e111275. doi: 10.1371/journal.pone.0111275 (PMC4206489; doi:10.1371/journal.pone.0111275)

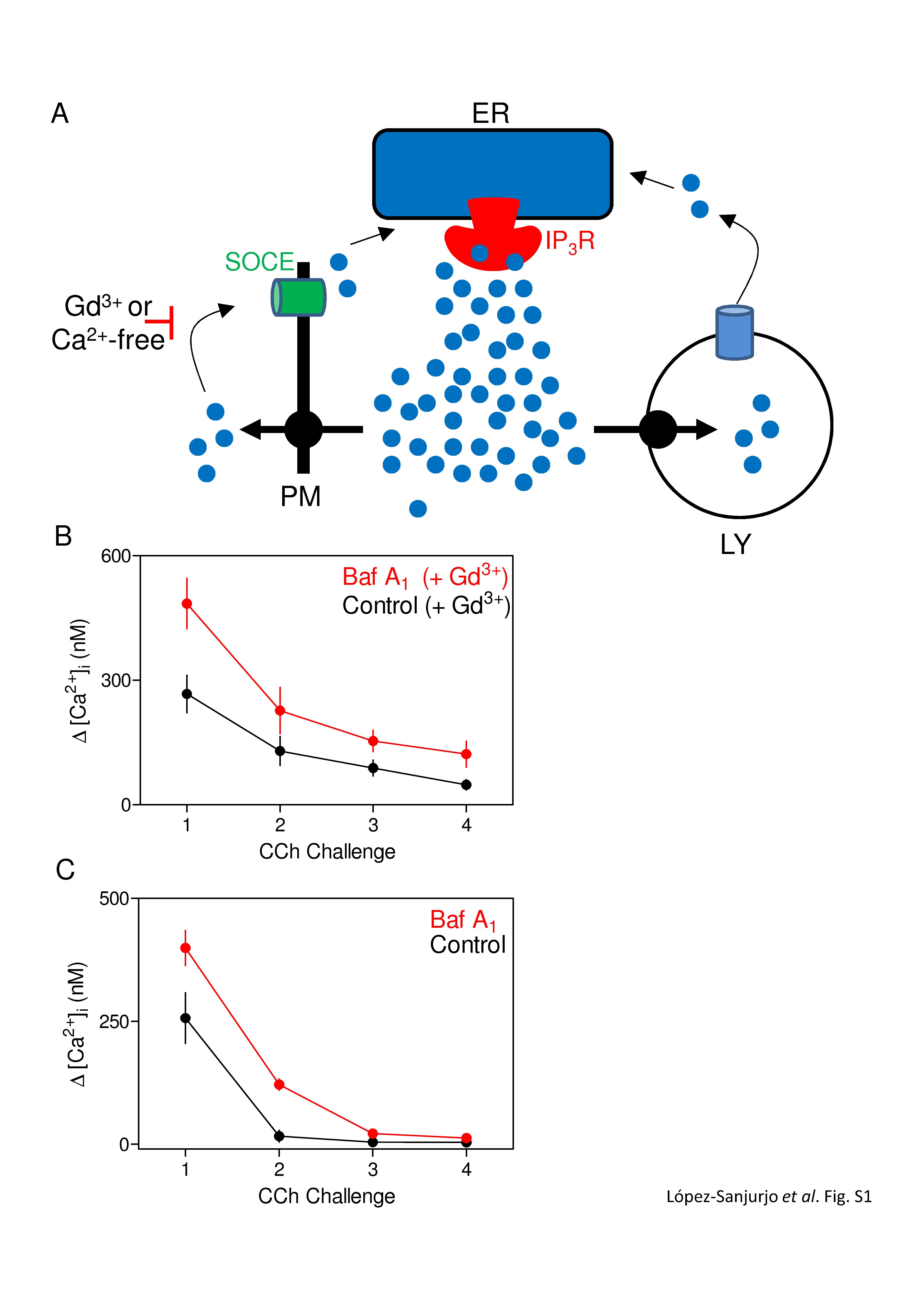

Supplement: Figure S1 — Responses to repetitive challenges with carbachol reveal that Ca2+ rapidly recycles from lysosomes. (A) A fraction of the Ca2+ released from the ER via IP3Rs is normally lost to the extracellular space as Ca2+ pumps in the plasma membrane (PM) extrude it from the cytosol. When Ca2+ is present in the extracellular medium, this loss is replenished by store-operated Ca2+ entry (SOCE). Removal of extracellular Ca2+ or blockade of SOCE by Gd3+ prevents this recycling of Ca2+. Lysosomes also sequester Ca2+ released by IP3Rs [30], but it is important to resolve whether that Ca2+ is also rapidly recycled via the cytosol to the ER. The experiments shown in Figure 5 address this issue. (B) The Ca2+ signals evoked by repetitive challenges with CCh (1 mM, 30 s) were recorded from HEK cells in Ca2+-free HBS with 1 mM Gd3+ (as shown in Figure 5B). The peak amplitudes of the Ca2+ signals are shown for control cells and cells treated with bafilomycin A1 (means ±S.E., n = 6). These raw data were used to produce Figure 5F. (C) Summary data (means ±S.E., n = 6) from experiments similar to those shown in (B), but in Ca2+-free HBS, show that in the absence of high concentrations of Gd3+, cells respond robustly to the first CCh challenge, but not to subsequent challenges. (TIF) [file pone.0111275.s001.tif]
